# Deciphering the Receptor Repertoire Encoding Specific Odorants by Time-Lapse Single-Cell Array Cytometry

**DOI:** 10.1038/srep19934

**Published:** 2016-02-02

**Authors:** Masato Suzuki, Nobuo Yoshimoto, Ken Shimono, Shun’ichi Kuroda

**Affiliations:** 1 Advanced Research Division, Panasonic Corporation, 3-4 Hikaridai, Seika, Kyoto 619-0237, Japan; 2 The Institute of Scientific and Industrial Research, Osaka University, 8-1 Mihogaoka, Ibaraki, Osaka 567-0047, Japan

## Abstract

Mammals can recognize a vast number of odorants by using olfactory receptors (ORs) known as G protein-coupled receptors. The *OR* gene family is one of the most diverse gene families in mammalian genomes. Because of the vast combinations of ORs and odorants, few ORs have thus far been linked to specific odorants. Here, we established a functional screening method for *OR* genes by using a microchamber array containing >5,400 single olfactory epithelium-derived cells from mice applied to time-lapse single-cell array cytometry. This method facilitated the prompt isolation of single olfactory sensory neurons (OSNs) responding to the odorant of interest. Subsequent single-cell RT-PCR allowed us to isolate the genes encoding respective ORs. By using volatile molecules recognized as biomarkers for lung cancers, this method could deorphanize ORs and thereby reconstitute the OR-mediated signaling cascade in HEK293T cells. Thus, our system could be applied to identify any receptor by using specific ligands in the fields of physiopathology and pharmacology.

The olfactory system of vertebrates can recognize a vast number of odorants^1^,^2^. Olfactory receptors (ORs) on olfactory sensory neurons (OSNs) play a central role in binding odorants and subsequently transducing biosignals. The ORs, belonging to the superfamily of G-protein-coupled-receptors (GPCRs)^3^,^4^, comprise 396 molecules in humans and 1,130 molecules in mice^5^,^6^. The OR family has been postulated to form repertoires for encoding specific odorants in a combinatorial manner (known as a “combinatorial receptor coding scheme”), which means that a single odorant molecule binds to the subset of ORs with different affinity, and a given OR is capable of recognizing multiple odorants^7^. For deciphering the OR repertoire encoding specific odorant, while the human OR repertoire has recently been analyzed by using heterologous cells expressing approximately 94% of all human ORs^8^, we were motivated to develop a high-throughput method for OR screening using native OSNs without any concern about chaperons or accessory proteins.

When screening ORs that respond to a specific odorant, native OSNs activated by odorants can be identified using Ca^2+^ imaging^9^. The interaction of an odorant with its OR enhances the intracellular cAMP concentration *via* heterotrimeric G-proteins (G_αolf_) and adenylyl cyclase type III, which leads to extracellular Ca^2+^ influx through the cyclic nucleotide-gated ion channel (CNG2)^10^. Consequently, the odorant-specific OSNs can be assigned to their respective ORs using a fluorescent Ca^2+^ indicator. A single OSN expresses only 1 type of OR on the surface of the olfactory cilia^11^; thus, the *OR* gene expressed in the isolated OSN can be cloned by single-cell reverse-transcription-polymerase chain reaction (RT-PCR). However, because an appropriate cell-to-cell distance needs to be maintained for manual single cell isolation using a glass capillary, the number of cells for Ca^2+^ imaging becomes too small (*i.e*., 50 ~ 100 cells in 1 experiment) to perform high-throughput screening.

To isolate target OSNs expressing an odorant-specific OR from a cell library, after stimulation with an odorant, it is necessary to measure the transient increase of intracellular Ca^2+^ concentration in each cell using time-resolved analysis for at least 5 min^7^. Additionally, it is known that the proportion of OSNs responding to 1 odorant is under 0.1% of olfactory epithelium-derived cells^12^,^13^. However, neither time-resolved analysis nor detection of target cells of less than 0.1% is available for conventional cell sorting technologies (*e.g*., fluorescence-activated cell sorter (FACS)). Recently, various single-cell analysis devices have been developed by using microwell arrays^14^,^15^,^16^. We also have developed an automated single-cell analysis and isolation system to facilitate high-throughput isolation of fluorescently labeled mammalian cells on a cell array in an undisruptive and single cell-based manner^17^. The robot is a stand-alone machine equipped with a microchamber array chip (up to ~2.5 × 10^5^ cells), an automated micromanipulator with a glass capillary, and a fluorescence microscope system, which could isolate single positive cells from ~2.5 × 10^5^ cells under undisruptive conditions. In this study, we have installed a perfusion system and a real-time calcium imaging system to the robot for stimulating cells with ligands and measuring extracellular calcium influx of each cell, respectively. The time-lapse single-cell array cytometry could analyze the extracellular calcium influx of ~5,400 olfactory epithelium-derived cells in a single-cell and time-resolved manner, and could retrieve positive single cells in an undisruptive manner for further analyses (functional assays, omics analyses, DNA sequencing).

## Results Section

### Development of Time-Lapse Single-Cell Array Cytometry

**Figure 1:**
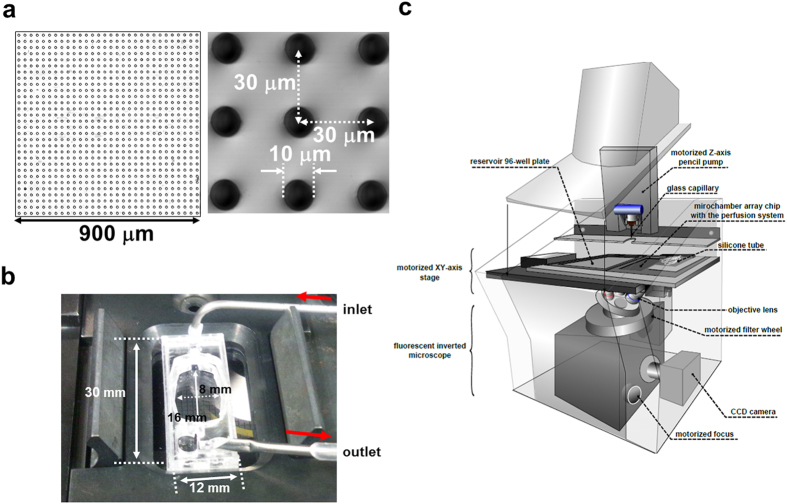
Components of time-lapse single-cell array cytometry. **(a)** The 10-μm polystyrene microchamber array chip. Magnified image of a subarea (900 wells, 30 × 30 wells; 900 × 900 μm, left) and micrograph of wells (10-μm diameter, 10-μm depth, 30-μm well-to-well pitch, right). Microchamber array chip contains 15 × 15 subareas (202,500 wells). **(b)** A perfusion system on a 10-μm microchamber array chip. Rhombus-shaped flow chamber (poly[methyl methacrylate], 16-mm length, 8-mm width, volume 204 μL). Ringer solution was circulated at 250 μL/min. **(c)** Automated single-cell analysis and isolation system. Details are described in the Methods section.

**Figure 2:**
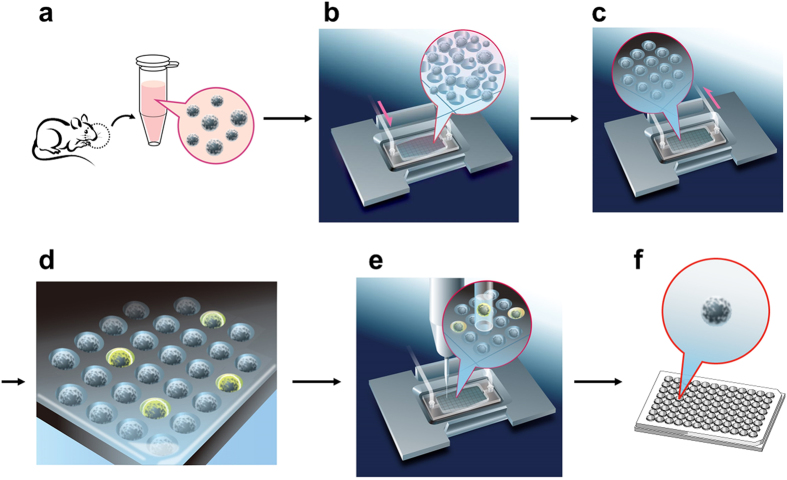
Schematic diagram of functional high-throughput screening system for OSNs responding to specific odorants. Mouse OE-derived cells were labeled with Fluo4-AM **(a)**, and applied to a 10-μm microchamber array chip equipped with a flow chamber, followed by brief centrifugation **(b)**. Floating and untrapped cells were removed by circulating Ringer solution and stimulated with an odorant **(c)**. OSNs expressing odorant-specific ORs showed transient fluorescence from the increase of intracellular Ca^2+^ concentration **(d)**. Each activated cell was retrieved **(e)**, transferred to the 96-well PCR plate **(f)**, and subjected to single-cell RT-PCR.

For screening *OR* genes from OSNs under physiological conditions, we planned to prepare the cell array of olfactory epithelium (OE)-derived cells, measure Ca^2+^ influx elicited by odorant stimulation, and retrieve responsive OSNs by an automated single-cell analysis and isolation system. The robot was vested with the following 3 key components to realize the time-lapse single-cell array cytometry analysis (Fig. 1): (i) A microchamber array chip containing 202,500 wells (10 μm diameter, Fig. 1a) for aligning single OE-derived cells at high density. (ii) An open perfusion system (Fig. 1b) for exchanging solutions on 57,600 wells of the chip continuously; the upper surface of the system is open for access of the glass capillary. (iii) An automated single-cell analysis and isolation system (Fig. 1c) for the acquisition of fluorescent intensity of each OSN on the microchamber array in a time-resolved manner, identification of OSNs responding to a specific odorant and automated isolation of assigned OSNs by glass capillary equipped on the micromanipulator. The robot can measure the odorant-elicited Ca^2+^ response of ~5,400 OE-derived cells containing more than 250 OSNs simultaneously. Candidate OSNs were automatically retrieved and then subjected to single-cell RT-PCR for identifying the *OR* genes expressed in each OSN (Fig. 2).

### Construction of OSN Array

**Figure 3:**
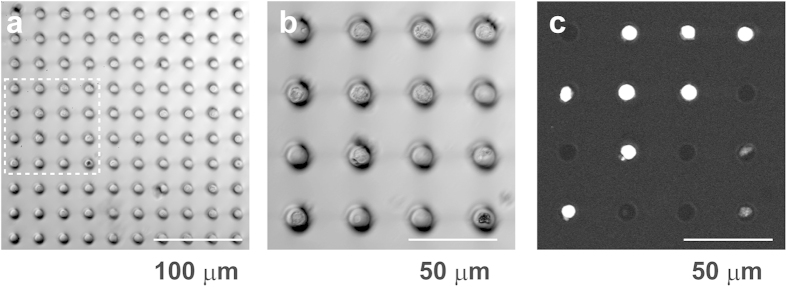
Introduction of olfactory epithelium-derived cells to 10-μm microchamber array chip. **(a)** Differential interference contrast (DIC) image of 10-μm microchamber array chip containing olfactory epithelium-derived cells. **(b)** Magnified image of dashed square in (**a**). **(c)** Fluorescent image of (**b**) labeled with Hoechst 33342. Single cells were entrapped in each single microchamber.

When mouse olfactory epithelium-derived cells (~4 × 10^5^ cells) were introduced into microchambers by brief centrifugation (7 × *g*, 1 min, 3 times) and subsequently labeled with Hoechst 33342, single cells were found to be entrapped in 30.0% ± 2.3% (N = 16) of 10-μm polystyrene microchambers under fluorescent microscopy (Fig. 3). A fluorescent LIVE/DEAD imaging method revealed that the viability of cells in 10-μm microchambers was 57.8% ± 5.4% (N = 4) (Fig. S1). Because the viability of cells prepared from mouse olfactory epithelium was 34.3% ± 2.7% (N = 8), the higher viability of cells in 10-μm microchambers may be attributed to a high sedimentation rate of live cells (hereafter named OSN array)

### Single Cell-level Calcium Imaging of OSN Array

**Figure 4:**
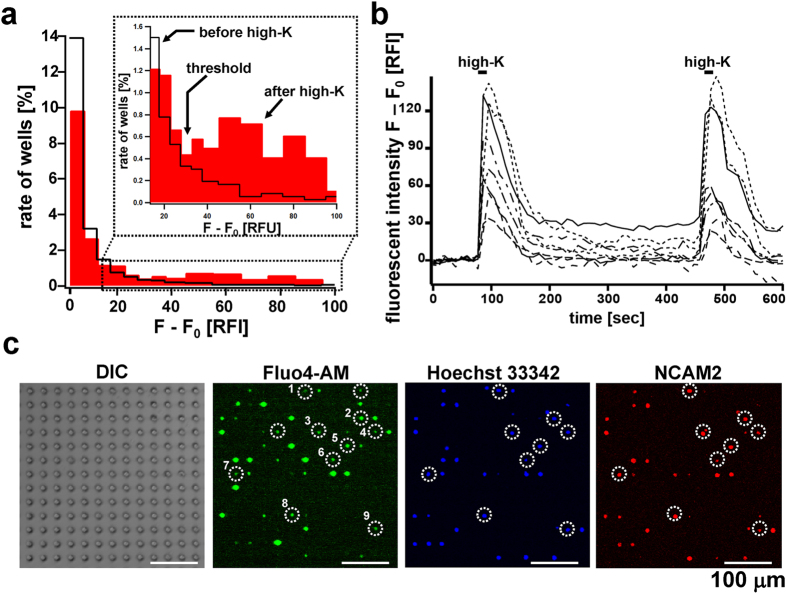
Ca^2+^ image of Fluo4-AM-labeled OSNs upon activation with high K solution. **(a)** Histogram of RFI changes (F−F_0_) of 5,400 microchambers. White bars, before high K treatment. Red bars, after high K treatment. Based on the RFI values before high K treatment, the threshold was set at F_0_ + 30. **(b)** Time course of relative fluorescence intensity (RFI) of the 9 marked OSNs. Addition of 100 mM KCl solution induced a dramatic but transient increase in Ca^2+^ response (F − F_0_). To examine if the Ca^2+^ channel (i.e., CNG2, cyclic nucleotide-gated ion channel) is active during measurement (600 sec), 100 mM KCl solution was added again to OSNs at 500 sec. **(c)** Micrographs of OSN array. DIC, differential interference contrast image of native OSNs; Fluo4-AM, fluorescent image of Fluo4-AM-labeled native OSNs; Hoechst 33342, nuclear staining image of fixed OSNs; and NCAM2, Alexa 569-labeled OSN-specific marker staining image of fixed OSNs. Bars, 100 μm. The nine cells marked with white circles (in the Fluo4-AM image) were activated by high K^+^ solution. Some cells showing strong fluorescent intensity (such as cells above and below #7 cell) were not the suprathreshold cells. Since OSNs possess neurites, single OSN was sometimes observed as two cells by NCAM2 staining, while confirmed as single cell by Hoechst 33342 staining (such as #3 cell). During the subsequent staining with NCAM2 and Hoechst 33342, some suprathreshold cells were accidentally washed out from microchambers (such as two cells in white circles without numbers in the Fluo4-AM image).

For the stimulation with potassium ion, the flow chamber was mounted on ~57,600 microchambers (polystyrene, 10-μm wells, 64 subareas) with silicone grease. Freshly prepared olfactory epithelium-derived cells (~4.0 × 10^5^ cells) were labeled with calcium indicator Fluo4-AM (acetoxymethyl form), added to the flow-chamber on microchamber array, and then centrifuged briefly to make an OSN array. After the cells were set into the robot, floating and untrapped cells were removed by circulating Ringer solution at 250 μL/min for 10 min. Relative fluorescent intensities (RFI) of each cell in 6 subareas (5,400 wells) were measured every 10 s for more than 5 min. First, relative fluorescent intensities of unstimulated cells (N = 748) were measured for 5 min. Based on the average (F_0_) and standard division (SD) of initial fluorescent intensities, the threshold was defined as F_0_ + 3 SD (F_0_ + 30 RFI), and the cells exhibiting more than F_0_ + 30 RFI were judged as positive cells. Because treatment with high-potassium solution (high-K solution, 100 mM KCl) elicits an increase in intracellular Ca^2+^ concentration in OSNs^7^, we observed the increase of fluorescent intensity (F−F_0_) after the exchange with high-K solution. As shown in Fig. 4a, 4.0% ± 0.7% (N = 5) of 5,400 microchambers showed significantly increased fluorescence intensity within 5 min (Movies S1 and S2). The rate of high-K-responsive cells in olfactory epithelium-derived cells was consistent with the rate published elsewhere (4.2–9.2%)^12^. The histogram of fluorescent intensity changes (F–F_0_) of the 5,400 microchambers upon addition of high-K solution showed the emergence of a broad peak, where the changes span from 30 to 100 (Fig. 4a), suggesting the threshold resides between the unstimulated cell-derived peak and the responsive cell-derived peak, namely (F–F_0_ = 30 RFI). Nine responsive cells of suprathreshold (F–F_0_) (Fig. 4b) were detected with an antibody against neural cell adhesion molecule 2 (NCAM2) known as a marker of OSNs located in zones 2, 3 and 4 at OE^18^,^19^, which confirmed that high-K-responsive cells are OSNs (Fig. 4c).

### Screening of ORs with Biomarkers for Lung Cancer

**Figure 5:**
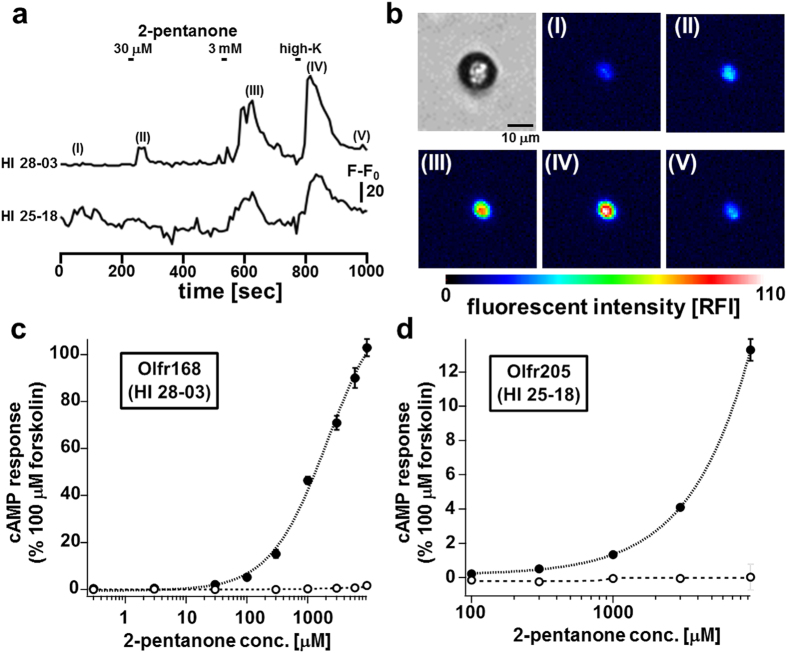
Functional high-throughput screening of 2-pentanone-responsive OSNs. **(a)** Time course of relative fluorescence intensity (RFI) of 2 Fluo4-loaded OSNs (IDs HI 28-03 and HI 25-18). I, Ringer solution; II, 30 μM 2-pentanone; III, 3 mM 2-pentanone; IV, 100 mM KCl; and V, Ringer solution. **(b)** Fluorescent images of OSN (ID HI28-03) at each step (I, II, III, IV, and V) of (**a**). The cAMP responses of HEK293T cells expressing Olfr168 **(c)** and Olfr205 **(d)**. The cAMP response elicited by 100 μM forskolin was defined as 100%. Closed circles, Olfr168/205-expressing cells; open circles, mock-transfected cells. Data represent the mean ± SEM (N = 12).

Since many volatile molecules in exhaled breath have been identified as biomarkers for various cancers^20^,^21^,^22^,^23^, ORs specific to each biomarker have been considered as promising sensing molecules for non-invasive diagnosis^24^,^25^. We therefore adopted the OSN array to isolate ORs that are specific to biomarkers for lung cancer, *i.e*., 2-pentanone, pyridine, and 2-butanone^26^,^27^. While measuring fluorescence continuously, the OSN array was treated with 30 μM 2-pentanone for 10 s, and subsequently with 3 mM 2-pentanone and 100 mM high K solution. Upon stimulation with 2-pentanone, a transient increase in fluorescent intensity occurred in 2 of 244 high K^+^ -responsive cells corresponding to 0.82% of high K-responsive cells (cell IDs, HI 28-03 and HI 25-18; Fig. 5a,b). The robot transferred these cells exhibiting Ca^2+^ responses to 2-pentanone and KCl to the 96-well PCR tubes individually with a glass capillary (Supplementary Fig. S2a). The overall success rate for cell transfer was 89.9% (in 69 trials). Then, these retrieved cells were subjected to single-cell RT-PCR using OR-specific PCR primers, which were designed to amplify the gene encoding common amino acid sequences of transmembrane segment 3 (TM3; containing a DRY motif) and TM7 in ORs (Supplementary Fig. S3)^9^. The overall success rate for single-cell RT-PCR was nearly 60% (in 40 trials) (Supplementary Fig. S2b). The 7 independent DNA fragments from HI 28-03 and HI 25-18 were found to be derived from the genes *Olfr168* (mOR271-1; GenBank accession number, AY317252) and *Olfr205* (mOR182-11 P; BC150839), respectively, corroborating the hypothesis that a single OSN expresses only 1 *OR* gene. After obtaining the full-length clones of *Olfr168* and *Olfr205* genes from mouse genomic DNA by PCR, HEK293T cells were transfected with expression vectors of each OR, G_αolf_, receptor-transporting protein 1 S (RTP1S), which aids the translocation of ORs from the Golgi to the cell membrane^28^, and a cAMP-sensing luciferase-based reporter^29^. The Olfr168-expressing cells showed an increase in luminescent intensity in response to 2-pentanone in a concentration-dependent manner, for which the EC_50_ was estimated at 2.1 ± 0.6 mM (N = 12) (Fig. 5c). Similarly, the Olfr205-expresing cells also responded to 2-pentanone in a dose-dependent manner, but to a lesser extent (Fig. 5d). These results demonstrated that our screening system provides a rational and reliable approach for deorphanizing ORs. Saito *et al*. previously deorphanized Olfr168 as a 2-pentanone-specific OR by manual functional screening, and estimated the EC_50_ to be below 10 μM^30^. Their lower EC_50_ of Olfr168 for 2-pentanone may be attributed to the difference in effector molecules used (cAMP-sensing luciferase-based reporter vs. protein kinase A-dependent reporter). Alternatively, they used Hana3A cells which may sensitize Olfr168 more effectively than HEK293T cells (HEK293T cells stably expressing RTP1L, RTP2, REEP1, and G_αolf_).

**Figure 6:**
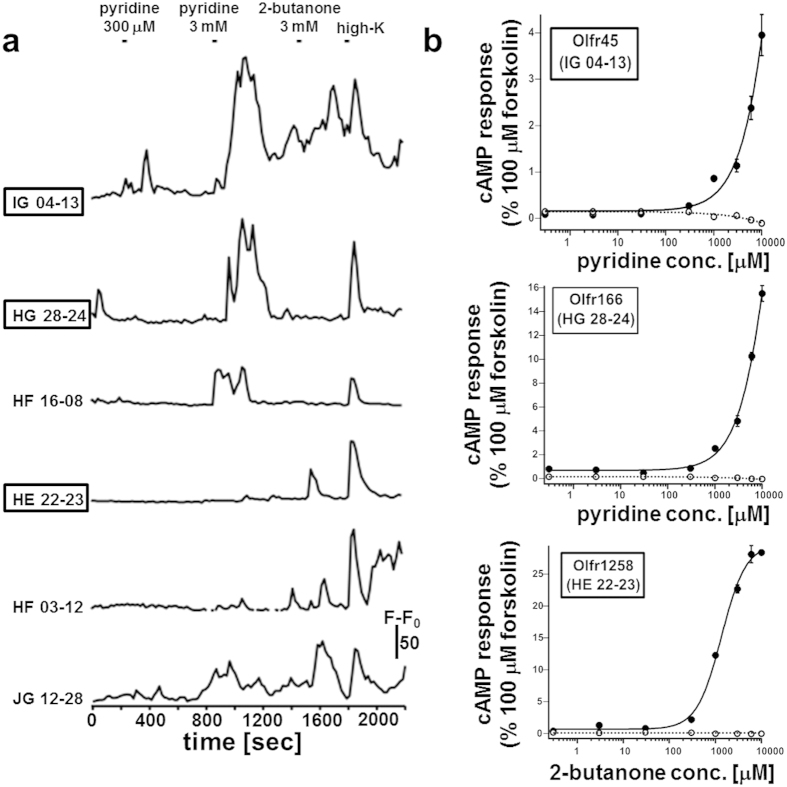
Functional high-throughput screening of ORs against pyridine and 2-butanone. (**a**) Time course of relative fluorescence intensity (RFI) of 6 Fluo4- loaded OSNs. Retrieved cells are indicated with boxes. (**b**) The cAMP responses of HEK293T cells expressing Olfr45, Olfr166, and Olfr1258. The cAMP response elicited by 100 μM forskolin was defined as 100%. Closed circles, OR-expressing cells; open circles, mock-transfected cells. Data represent the mean ± SEM (N = 12).

Next, we applied the screening system for discovering ORs responsive to pyridine and 2-butanone. Among the 239 high K-responsive cells, 3 cells (IDs IG 04-13, HG 28-24, and HF 16-08) were found to respond against only pyridine, 2 cells (IDs HE 22-23 and HF 03-12) were responded to only 2-butanone, and 1 cell (ID JG 12-28) was responded to both odorants (Fig. 6a, Supplementary Video S3 for HG 28-24). Two cells (IG 04-13 and HG 28-24) of four pyridine-responsive cells were retrieved (Supplementary Fig. S2a), and then found to express Olfr45 (mOR253-2; GenBank accession number, AY317653) and Olfr166 (mOR270-1; AY317250), respectively. One cell (HE 22-23) of three 2-butanone-responsive cells expressed Olfr1258 (mOR232-3; AY318460). These ORs were similarly expressed in HEK293T cells and then examined if they elicit cAMP response upon odorant stimulation. As shown in Fig. 6b, both Olfr45 and Olfr166 were activated by pyridine, and Olfr1258 was activated by 2-butanone. Thus, an odorant of interest could be used to directly isolate the ORs comprising its respective repertoire from the pool of orphan ORs in a high-throughput manner. However, among the 24 ORs isolated by stimulation with the 4 odorants used in this study, only 6 ORs showed responses to their respective odorants in HEK293T cells. When the cAMP responses in HEK293T cells were compared with the cAMP-dependent Ca^2+^ responses in native OSNs, all ORs that were successfully expressed in HEK293T cells (Olfr168, Olfr45, Olfr166, Olfr205, and Olfr1258) required higher concentrations of their respective odorants than did endogenous ORs in OSNs (Fig. 5c,d and 6b). These results may be attributed to the differences between HEK293T cells and OSNs in following issues: effector molecules for ORs, sensitivity of cytometric methods, expression level of ORs, and surface display efficiency of ORs. For examples, as reported previously, the expression efficiency may be improved by co-expressing either receptor-transporting proteins^28^, other GPCRs^31^, other RTPs besides RTP1S^30^, or guanine nucleotide exchange factor Ric-8 A (for sensitizing Olfr)^32^. Otherwise, the CNG2 channel in OSNs may be more sensitive to cAMP compared to the cAMP-sensing luciferase-based reporter in HEK293T cells.

## Discussion Section

In this study, we performed high-throughput functional screening of OR genes from native OSNs by using the time-lapse single-cell array cytometry equipped with a perfusion system and an automated single cell retrieval system, and finally deorphanized 5 ORs responding to biomarker molecules for lung cancer (Olfr168 and Olfr205 for 2-pentanone, Olfr45 and Olfr166 for pyridine, and Olfr1258 for 2-butanone). These results demonstrated, for the first time, that the combinatorial receptor-coding scheme of ORs in OE could be partly reconstituted on a microchamber array. For comprehensive decipherment of OR repertoires to specific odorants, theoretically more than 35 sets of an OSN array containing ~250 OSNs have to be screened by the robot in order to cover the full set of OR molecules (*e.g*., 1,130 in the mouse). Thus, it was demonstrated that the robot is suitable for the decipherment of OR repertoires to specific odorants by using native OSNs. In addition to functional OR cloning using OSNs, this cloning strategy could be applicable to heterologous cells expressing whole ORs from humans and mice, and would facilitate the isolation of other receptors including taste GPCRs, transporters, and ion channels responsible for detection of orphan ligands.

The odorants used in this study (2-pentanone, 2-butanone, and pyridine) were known as volatile biomarkers for lung cancer^26^,^27^. Since other volatile biomarkers were also found in other cancers, these biomarkers in blood, saliva, urine, skin, and exhaled breath are considered as promising target molecules for non-invasive diagnosis^33^. However, conventional sensing molecules (e.g., antibodies, enzymes, aptamers, and molecular imprinting) are not applicable for detecting small molecules such as volatile compounds in a highly specific manner. These situations have led us to utilize ORs as sensing molecules for the diagnosis of cancers. As demonstrated in all ORs examined, the OR expressed in exogenous expression system showed too insufficient sensitivity to detect biomarkers in specimen. For instance, while Olfr168 expressed in HEK293T cells could detect more than 100 μM 2-pentanone, the average concentration of 2-pentanone in urine from lung cancer patient is 1.9 μM^27^. It is indispensable to sensitize ORs in exogenous expression system for measuring biomarkers by using their cellular functions. Alternatively, the physical interaction of ORs with odorants could be used for highly sensitive detection of biomarkers, because the OR-coupled carbon nanotube-based field effect transistor detected volatile molecules in gas at ppm order and in liquid at 1 pM order^25^,^34^. Another concern about the use of ORs is broad odorant specificity which is sometimes observed in certain ORs (as shown in JG 12-28). We should carefully choose ORs to maximize the specificity to odorant of interest.

## Methods Section

### Automated single-cell analysis and isolation system

The chip (polystyrene; diameter of wells, 10 μm; depth, 10 μm; well-to-well pitch, 30 μm; 30 × 30 wells/subarea; 15 × 15 subareas/chip; 202,500 wells/chip) was purchased from As One Corp. (Osaka, Japan). Detailed specifications are described in Fig. 1. The flow chamber (rhombus-shaped flow chamber; length, 16 mm; width, 8 mm; volume, 204 μL) was made from poly(methyl methacrylate) (PMMA) by using CO_2_ laser processing (Versa Laser VLS 2.30; Universal Laser Systems, Inc., Scottsdale, AZ). The inlet and outlet of the flow chamber were connected to a peristaltic pump with silicon tubing to circulate the solution. Detailed specifications are shown in Fig. 1B. The flow chamber was mounted on the washed 10-μm polystyrene microchamber array chip with silicon grease.

The automated single-cell analysis and isolation system was described previously^17^, which is commercially available from As One Corp. (Osaka, Japan). Briefly, as shown in Fig. 1c, the system is composed of a fluorescent inverted microscope, a CCD camera, a precision motorized XY-axis stage, a motorized Z-axis pencil pump equipped with a glass capillary (internal diameter, ~12 μm; outer diameter, ~15 μm), and a reservoir 96-well plate. The capillary was prepared from a glass tube (internal diameter, 0.86 mm; outer diameter, 1.5 mm; GC150F-10; Harvard apparatus; Kent, UK) by using a capillary puller (P-97/IVF; Shutter Instruments Co.; Novato, CA). The system could measure changes in the fluorescent intensity of >5,400 wells at 10-s intervals continuously, select cells based on the change in fluorescent intensity, and automatically transfer each selected cell from microchambers to a 96-well PCR plate with a glass capillary at a rate of ~15 s per cell.

### Preparation of OSNs array

All animal experiments were conducted according to the Osaka University guidelines for the care and use of animals. All animal experiments were approved by the Committee for Animal Experiments of Osaka University. After induction of anesthesia by an intraperitoneal injection of sodium pentobarbital (Kyoritsu Seiyaku Corp.; Tokyo, Japan), olfactory epithelium (OE) tissues were isolated from 3- to 5-week-old female C57BL/6 J mice (Japan SLC Inc.; Hamamatsu, Japan) and dissected in iced Ca^2+^ -free Ringer solution (140 mM NaCl, 5 mM KCl, 10 mM HEPES, 1 mM EDTA, 10 mM glucose, and 1 mM sodium pyruvate [pH 7.2]). The OE tissues were minced using scissors and treated with 1 U/mL papain (Sigma-Aldrich; St. Louis, MO, USA) in Ca^2+^ -free Ringer solution containing 1 mM Cys (Sigma-Aldrich) at 37 °C for 45 min with gentle rotation. To stop the enzymatic reaction, Ringer solution (140 mM NaCl, 5 mM KCl, 1 mM CaCl_2_, 1 mM MgCl_2_, 10 mM HEPES, 10 mM glucose, and 1 mM sodium pyruvate [pH 7.2]) containing 0.1 mg/mL DNase I (Worthington Biochemical Corp.; Lakewood, NJ, USA), 0.1 mg/mL bovine serum albumin, and 500 μM leupeptin (Sigma-Aldrich) was added. The cell mixture was passed through a cell strainer (35-μm mesh; Becton Dickinson Corp.; Franklin Lakes, NJ, USA) twice, subjected to centrifugation at 700 × *g* for 5 min twice, and resuspended in Ringer solution that had been prewarmed at 37 °C. Following trypan blue staining, the OE-derived cells were counted with a TC10 cell counter (Bio-Rad Laboratories, Inc.; Hercules, CA, USA). For calcium imaging, ~4 × 10^5^ cells were applied to a 10-mm polystyrene microchamber array chip equipped with a flow chamber, incubated with 5 μM Fluo4-AM (acetoxymethyl ester) (Dojindo Molecular Technologies Inc.; Kumamoto, Japan), and 0.02% (w/v) Pluronic F-127 (Life Technologies Corp.; Carlsbad, CA, USA) at 37 °C for 20 min, and then subjected to centrifugation at 7 × *g* at room temperature for 1 min 3 times. The chip was set in the automated single-cell analysis and isolation system, washed with circulating Ringer solution at room temperature (250 μL/min) for 10 min to remove floating and untrapped cells, and then promptly used for the analysis.

### Cytochemical analyses of OSNs

To measure the viability of OSNs in the microchamber array, cells without Fluo4-AM treatment were incubated with 1 μM calcein-AM (Dojindo Molecular Technologies Inc.) and 2.25 μM propidium iodide (PI) (Dojindo Molecular Technologies Inc.) at room temperature for 30 min in a dark environment. After the OSN array was washed 2 times with phosphate-buffered saline (PBS), fluorescent images were obtained with an inverted fluorescent microscope (IX-81; Olympus Corp.; Tokyo, Japan). To confirm the positions of OSNs in the array, cells were fixed with 4% (w/v) paraformaldehyde in PBS at room temperature for 30 min, washed 2 times with PBS, immersed in 10% (v/v) goat serum (Sigma-Aldrich) at room temperature for 60 min, reacted with 2 μg/mL anti-neural cell adhesion molecular 2 (anti-NCAM2) rabbit polyclonal IgG antibody (Santa Cruz Biotechnology Inc.; Santa Cruz, CA, USA) in PBS containing 1% goat serum at room temperature for 60 min, and finally labeled with 1 μg/mL Alexa Fluor 568-labeled anti-rabbit IgG (Life Technologies Corp.) and 1 μg/mL Hoechst 33342 (Life Technologies Corp.) at room temperature for 60 min.

### Screening of OSNs expressing ORs to odorants of interest

Although the automated single-cell analysis and isolation system was able to determine the fluorescent intensities of 225 subareas (containing 202,500 wells), because of the upper limit of the system’s processing speed, only 6 subareas (5,400 wells) were used to measure fluorescent intensity at 10-s intervals. To measure F_0_, Ringer solution was circulated for the first 5 min. For stimulation with the odorant, the solution was switched to odorant solution for 10 s and then returned to Ringer solution for at least 5 min. The odorant solutions were freshly prepared by diluting 3 M odorant stocks (in dimethyl sulfoxide) with Ringer solution. Finally, for induction of the maximum Ca^2+^ response, a high-K solution (100 mM KCl in Ringer solution) was used for 10 s. After the measurement, the change in fluorescence intensity (F – F_0_) value for each odorant was calculated for each cell. Cells exhibiting values higher than 30 were automatically judged as positive cells. Finally, odorant-positive and high-K-positive cells were automatically assigned and retrieved from the microchambers through a glass capillary whose tip was filled with Ringer solution. The retrieved single cell in the glass capillary (approximately 50 nL) was ejected into the well of a full-skirt type 96-well PCR plate containing 4.5 μL cell lysis solution from the single-cell RT-PCR kit (CellAmp Whole Transcriptome Amplification Kit Ver. 2; Takara Bio Inc.; Shiga, Japan).

### Single cell RT-PCR

The single OSN-derived cDNA library was used as a template to amplify *OR* genes by using degenerate PCR primer pairs, which were designed to anneal the nucleotide sequences encoding common amino acid sequences of TM3 and TM7 within ORs. The forward primer TM3 (5′-ATGGCITAYGAYMGITAYGTIGC-3′), the reverse primer II-4 (5′-TCICGRTTYCTIAGRCTRTAIATRAAIGGRTT-3′), and the reverse primer II-5 (5′-TCYTTRTTYCTIAGRCTRTAIATIATIGGRTT-3′) were synthesized (Sigma-Aldrich) (Supplementary Fig. S3). The TM3 primer is based on the published nucleotide sequence^9^. The II-4 and II-5 primers were based on the same amino acid sequence of ORs, as reported previously^7^. The PCR was performed using a DNA Engine Cycler (PTC-200; Bio-Rad Laboratories Inc.) with 0.05 U/mL LA-Taq (Takara Bio) in 25 μL of GC buffer I (from LA-Taq kit) containing 0.4 mM dNTPs, 1.2 μM primer pairs, and 0.1 μL of the single OSN-derived cDNA library. The PCR schedule was 94 °C for 1 min, 1 cycle; 94 °C for 0.5 min, 40 °C for 0.5 min, and 72 °C for 2 min, 35 cycles; and 72 °C for 5 min, 1 cycle. The PCR products were subcloned into the pMD20 vector (Takara Bio), followed by sequencing analysis. All recombinant DNA experiments were conducted according to the Osaka University guidelines for the recombinant DNA experiments. All recombinant DNA experiments were approved by the Committee for Recombinant DNA Experiments of Osaka University.

### Cloning of OR, G_αolf_, and RTP1S genes

The full-length sequences of *Olfr45*, *Olfr166*, *Olfr168, Olfr205* and *Olfr1258* genes were amplified by PCR with LA-Taq from mouse genomic DNA. These PCR products were subcloned into the pCI-neo vector (Promega; Madison, WI, USA), which is used for the mammalian expression of N-terminally rhodopsin-tagged and myc-tagged exogenous protein. The former tag is known to enhance the translocation of ORs to the cell membrane^35^. The *G*_*αolf*_^36^ and *RTP1S*^37^ genes were both cloned from mouse genomic DNA by PCR and inserted into the pCI-neo vector.

### Cell-based functional assay

Human embryonic kidney HEK 293 T cells were grown in Dulbecco’s modified Eagle medium supplemented with 10% (v/v) fetal bovine serum, 100 U/mL penicillin, and 100 μg/mL streptomycin at 37 °C under humidified conditions containing 5% (v/v) CO_2_. The cells (1 × 10^4^ cells/well) were seeded in a 96-well white cell culture plate (Becton Dickinson Corp.). After 24 h, the cells were transfected using 0.5 μL of Lipofectamine 2000 (Life Technologies) with 0.3 μg of OR-expression plasmid and 0.1 μg of pGlosensor-22 F (Promega) encoding a cAMP-sensing luciferase-based reporter^20^, 0.1 μg of G_αolf_-expression plasmid, or 0.1 μg of RTP1S-expression plasmid. After 40 ~ 50 h, the endogenous level of luciferase activity was measured with a luminometer (GloMax-Multi; Promega) using the GloSensor cAMP assay (Promega), which was defined as the control luminescence value (L_0_). Upon stimulation with odorant, the luciferase activity induced was defined as the luminescence of the sample (L). Finally, the maximum level of luminescence (L_max_) was obtained by the addition of 100 μM forskolin. Each measurement was performed for at least 30 min. The odorant-dependent change in intracellular cAMP concentrations was expressed as [(L − L_0_)/(L_max_ − L_0_)] × 100.

## Additional Information

**How to cite this article**: Suzuki, M. *et al*. Deciphering the Receptor Repertoire Encoding Specific Odorants by Time-Lapse Single-Cell Array Cytometry. *Sci. Rep*. **6**, 19934; doi: 10.1038/srep19934 (2016).

## Supplementary Material

Supplementary Information

Supplementary Movie S1

Supplementary Movie S2

Supplementary Movie S3
