# Supplementary material for: Deciphering the Receptor Repertoire Encoding Specific Odorants by Time-Lapse Single-Cell Array Cytometry
Source: Sci Rep. 2016 Feb 2;6:19934. doi: 10.1038/srep19934 (PMC4735795; doi:10.1038/srep19934)
Supplement: Supplementary Information [file srep19934-s1.pdf]

Deciphering the Receptor Repertoire Encoding Specific Odorants by Time-Lapse Single-Cell Array Cytometry

Author names

Masato Suzuki[1], Nobuo Yoshimoto[2], Ken Shimono[1,*] & Shun'ichi Kuroda[2,*]

Affiliations

[1] Advanced Research Division, Panasonic Corporation, 3-4 Hikaridai, Seika, Kyoto 619-0237, Japan;

[2] The Institute of Scientific and Industrial Research, Osaka University, 8-1 Mihogaoka, Ibaraki, Osaka 567-0047, Japan.

Correspondence and requests for materials should be addressed to K. S. (shimono.ken@jp.panasonic.com) & S. K. (skuroda@sanken.osaka-u.ac.jp).

Supplementary Information

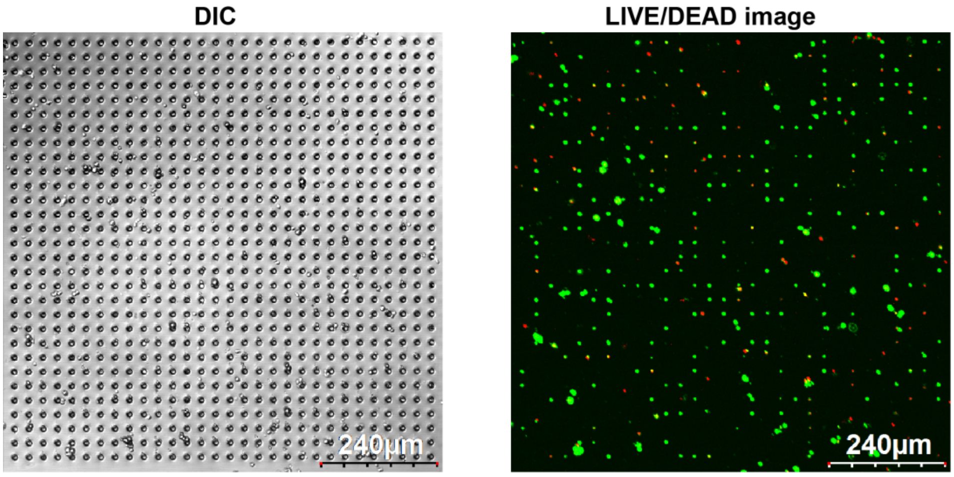

Supplementary Figure S1. Live/Dead fluorescent image of olfactory epithelium-derived cells in a 10-µm microchamber array chip. Live and dead cells were stained with Calcein-AM (acetoxymethyl form, green) and PI (propidium iodide, red), respectively.

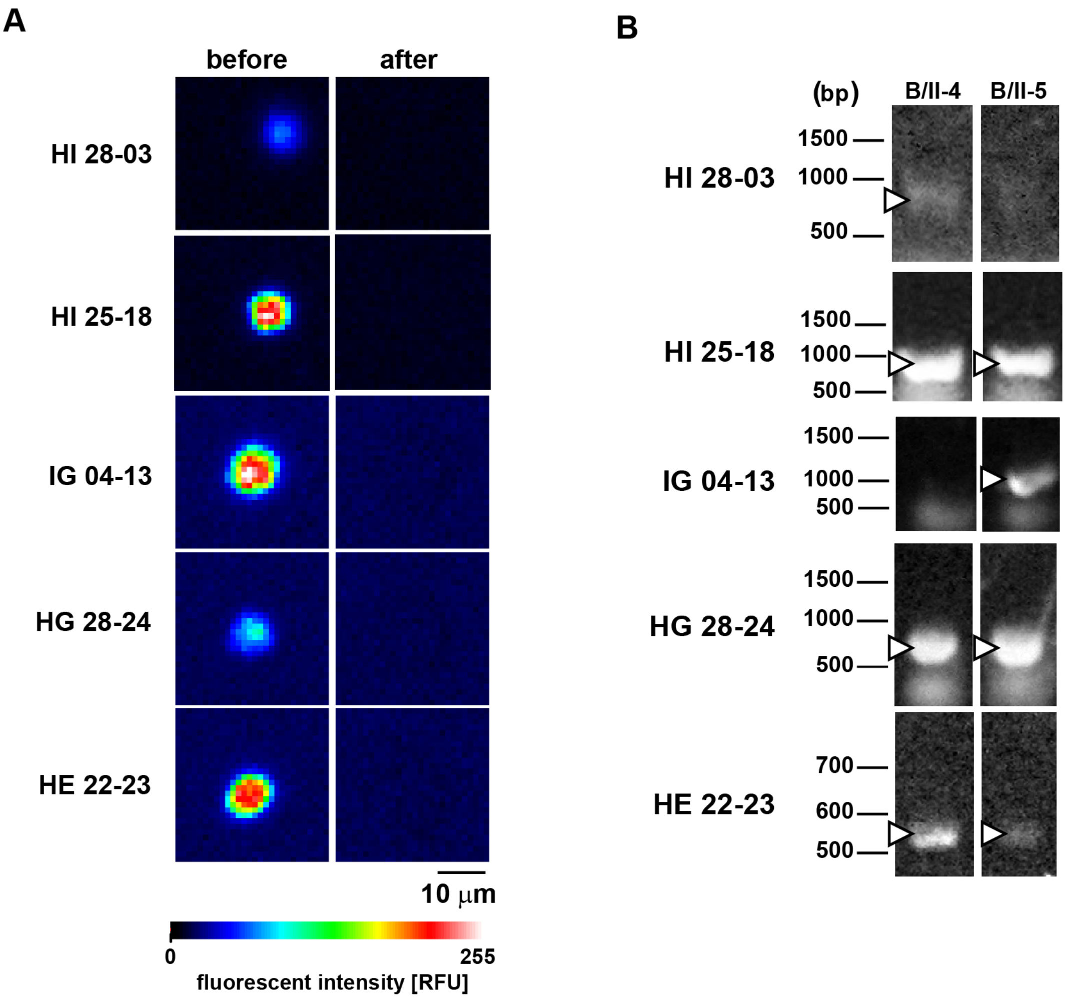

Supplementary Figure S2. Ca^{2+} images of native OSNs in a 10-µm microchamber array chip. (a) Fluorescent images of 5 selected OSNs (HI 28-03, HI 25-18, IG 04-13, HG 28-24, and HE 22-23) were captured before and after retrieval. **(b)** Agarose gel electrophoresis of single-cell PCR products of 5 selected OSNs. DNA sizes (bp, base pairs) are indicated in the left margin. Two sets of primers (B/II-4 and B/II-5) were used. White arrowheads indicate the expected amplicons.

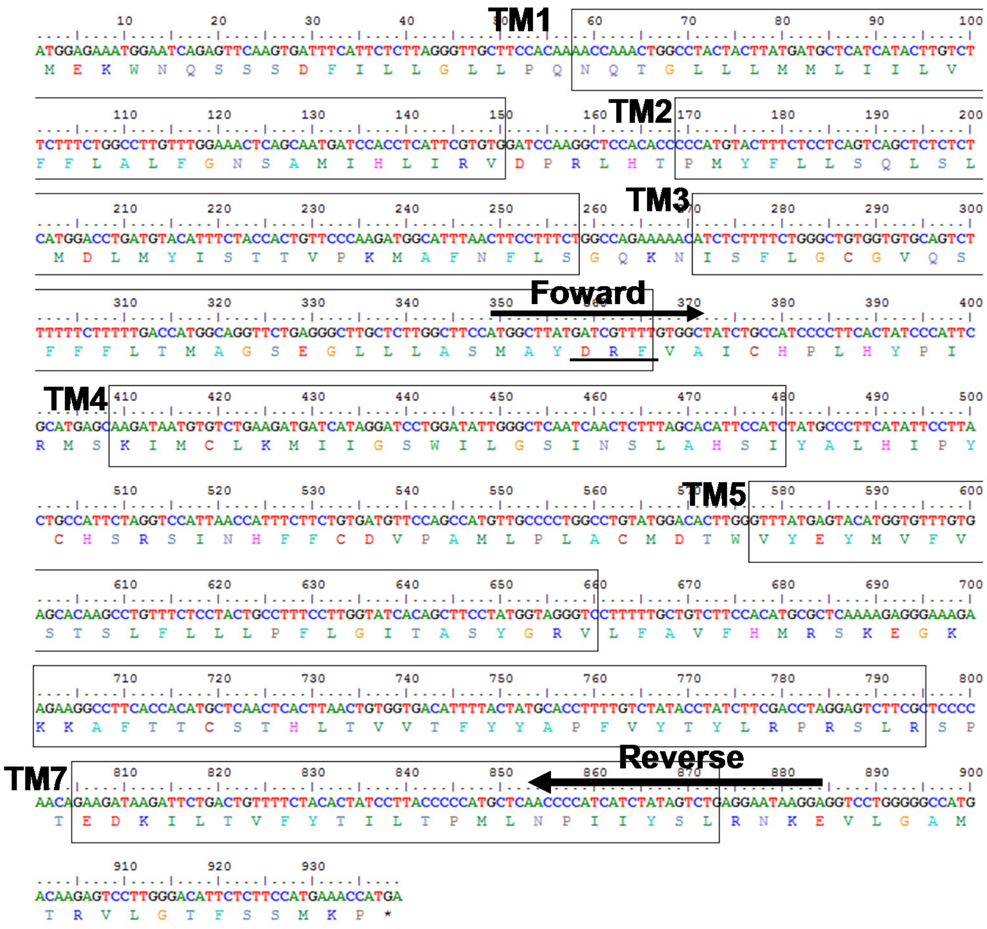

Supplementary Figure S3. Nucleotide sequence of the *Olfr168* gene. Primers used for PCR are indicated by black arrows. Transmembrane (TM) segments are indicated with boxes. The DRY motif in TM segment 3 is underlined.

Supplementary video 1. Time-resolved Ca^{2+} imaging analyses of 8 OSNs in 10-μm microchambers by using an automated single-cell analysis and isolation system. Cells were activated with high-K solution.

Supplementary video 2. Time-resolved Ca^{2+} imaging analyses of OSNs in 1 subarea (900 wells) of 10-μm microchambers performed using an automated single-cell analysis and isolation system. Cells were activated with high-K solution.

Supplementary video 3. Time-resolved Ca^{2+} imaging analyses of an OSN (ID HG 28-24) in a 10-μm microchamber by using an automated single-cell analysis and isolation system. Cells were activated with 3 mM pyridine.
